# Abnormality in glutamine–glutamate cycle in the cerebrospinal fluid of cognitively intact elderly individuals with major depressive disorder: a 3-year follow-up study

**DOI:** 10.1038/tp.2016.8

**Published:** 2016-03-01

**Authors:** K Hashimoto, D Bruno, J Nierenberg, C R Marmar, H Zetterberg, K Blennow, N Pomara

**Affiliations:** 1 Division of Clinical Neuroscience, Chiba University Center for Forensic Mental Health, Chiba, Japan; 2 Department of Psychology, Liverpool Hope University, Liverpool, UK; 3 Nathan S. Kline Institute for Psychiatric Research, Orangeburg, NY, USA; 4 Department of Psychiatry, New York University Langone Medical Center, New York, NY, USA; 5 Clinical Neurochemistry Laboratory, Institute of Neuroscience and Physiology, The Sahlgrenska Academy at the University of Gothenburg, Mölndal, Sweden; 6 Department of Molecular Neuroscience, UCL Institute of Neurology, London, UK

## Abstract

Major depressive disorder (MDD), common in the elderly, is a risk factor for dementia. Abnormalities in glutamatergic neurotransmission via the *N*-methyl-d-aspartate receptor (NMDA-R) have a key role in the pathophysiology of depression. This study examined whether depression was associated with cerebrospinal fluid (CSF) levels of NMDA-R neurotransmission-associated amino acids in cognitively intact elderly individuals with MDD and age- and gender-matched healthy controls. CSF was obtained from 47 volunteers (MDD group, *N*=28; age- and gender-matched comparison group, *N*=19) at baseline and 3-year follow-up (MDD group, *N*=19; comparison group, *N*=17). CSF levels of glutamine, glutamate, glycine, l-serine and d-serine were measured by high-performance liquid chromatography. CSF levels of amino acids did not differ across MDD and comparison groups. However, the ratio of glutamine to glutamate was significantly higher at baseline in subjects with MDD than in controls. The ratio decreased in individuals with MDD over the 3-year follow-up, and this decrease correlated with a decrease in the severity of depression. No correlations between absolute amino-acid levels and clinical variables were observed, nor were correlations between amino acids and other biomarkers (for example, amyloid-β_42,_ amyloid-β_40_, and total and phosphorylated tau protein) detected. These results suggest that abnormalities in the glutamine–glutamate cycle in the communication between glia and neurons may have a role in the pathophysiology of depression in the elderly. Furthermore, the glutamine/glutamate ratio in CSF may be a state biomarker for depression.

## Introduction

Late-life depression, one of the most common psychiatric disorders in older adults, is associated with significant functional impairment, variable treatment response, high recurrence rates, chronicity and high rates of medical comorbidity and mortality.^1, 2^ Multiple lines of evidence suggest that late-life depression is a risk factor for the development of dementia, including Alzheimer's disease (AD) and vascular dementia.^2, 3, 4^ However, the precise molecular mechanisms underlying the relationship between late-life depression and dementia risk remain unknown. Better understanding this relationship would likely contribute to improving preventive interventions in the elderly.

Glutamate (or l-glutamic acid) has an important role as the major excitatory neurotransmitter in the central nervous system. In the brain, glutamate is synthesized from glutamine by glutaminase, and metabolized to glutamine by glutamine synthetase (GS) in astrocytes.^5, 6, 7, 8^ Accumulating evidence suggests that abnormalities in the glutamatergic neurotransmission via the *N*-methyl-d-aspartate receptor (NMDA-R) have a key role in the pathophysiology of major depressive disorder (MDD).^5, 6, 8, 9, 10, 11, 12^ Hashimoto *et al.*^13^ reported increased levels of glutamate in the prefrontal cortex in postmortem brain samples from MDD and bipolar disorder, suggesting a role of the glutamatergic system in mood disorders. A noninvasive *in vivo* proton magnetic resonance spectroscopy (^1^H-MRS) study revealed increased levels of glutamate in the occipital cortex of patients with MDD,^14^ although other ^1^H-MRS studies found decreased levels of Glx (glutamate and glutamine) in the anterior cingulate cortex^15^ and dorsomedial/dorsal anterolateral prefrontal cortex^16^ in patients with MDD. In contrast, there was no difference in glutamate levels in the occipital cortex between MDD patients and controls.^17^ A recent MRS study showed that young MDD (*N*=90) and bipolar disorder (*N*=75) groups (18–30 years) did not show different levels of hippocampal glutamate compared with controls (*N*=40).^18^ A recent meta-analysis showed a significant reduction in Glx levels, but not glutamate levels alone, in the brains of depressed patients compared with controls across the studies included.^19^

Levine *et al.*^20^ reported higher cerebrospinal fluid (CSF) levels of glutamine in unmedicated patients with MDD. In contrast, a study showed low glutamate levels in the CSF of refractory patients with affective disorder.^21^ A recent study showed no significant differences between the control (*N*=25) and the MDD groups (*N*=18) in baseline CSF levels of glutamate (*P*=0.761) and glutamine (*P*=0.226).^22^ Thus, the results of studies on CSF levels of glutamine and glutamate in MDD patients are inconsistent. At present, there is no report that has evaluated the ratio of glutamine to glutamate in CSF of MDD patients although this ratio is an index of glutamine–glutamate cycle in glia–neuron communication.^5, 6, 8, 23, 24^ As the abnormalities in NMDA-R neurotransmission by altered glutamine–glutamate cycle in the brain may be involved in the pathophysiology of MDD,^5, 6^ it is of great interest to examine CSF levels of amino acids related with NMDA-R in elderly patients with MDD, and age- and gender-matched healthy controls.

In the present study, we measured CSF levels of amino acids (for example, glutamate, glutamine, glycine, d-serine and l-serine) in elderly patients with MDD, and age- and gender-matched healthy controls. All these amino acids can affect glutamatergic neurotransmission via the NMDA-R.^13, 25^ Furthermore, we also measured 3-year follow-up CSF samples in these subjects.

## Materials and methods

### Participants

This study was approved by the institutional review boards of the Nathan Kline Institute for Psychiatric Research and the New York University School of Medicine. Participants were volunteers who responded to advertisements in local newspapers and flyers, or were recruited from the Memory Education and Research Initiative Program.^26^ All participants provided informed consent before examination, and received up to $450.00 in compensation. A total of 133 participants completed the baseline evaluation, and 51 of these took part in the optional lumbar puncture procedure. Of these 51 participants, 3 were excluded because of evidence in their magnetic resonance imaging scans of confluent deep or periventricular white matter hyperintensities, defined as one or more hyperintense lesions measuring at least 10 mm in any direction. One individual was excluded because of a Mini-Mental State Examination (MMSE) score below 28. Of the 47 remaining participants, 28 were diagnosed with MDD by a board-certified psychiatrist, leaving 19 comparison subjects. The Structural Clinical Interview for the Diagnostic and Statistical Manual of Mental Disorders (DSM)-IV disorders (SCID) was administered by a psychiatrist to establish an MDD diagnosis. Of the 28 individuals with MDD, 21 (75%) had recurrent episodes. Table 1 summarizes the demographic and clinical characteristics of the study participants at baseline.

### Procedure

The study was conducted over four visits, usually 1 week apart. The first three visits were conducted at the Nathan Kline Institute for Psychiatric Research and the Clinical and Translational Science Institute, New York University Langone Medical Center. During the first visit, for the purpose of obtaining informed consent, study procedures were explained and participants were informed of their rights. Participants' medical and psychiatric histories, including family history of AD, were also obtained, and their vital signs were measured. Participants then underwent a psychiatric evaluation, and their global cognitive status was assessed using the MMSE. In addition, the Hamilton Depression Rating Scale (HAM-D) was administered to rate the severity of current depressive symptoms. Subjects who met the criteria for past MDD but were not currently depressed (that is, HAM-D score below 16) were included as MDD subjects. Blood was drawn for routine medical testing and *APOE* genotyping. During the second visit, participants underwent a magnetic resonance imaging scan of the head to quantify the magnitude of vascular brain pathology. During the third visit, subjects underwent a comprehensive neuropsychological assessment, including the Buschke Selective Reminding Test,^27^ the Trail-Making Test parts A and B,^28^ and the category fluency test.^29^

Finally, during the fourth visit, a lumbar puncture was performed by a neuroradiologist under guided fluoroscopy in a subset of participants. Before the procedure, which was performed between 0900 and 1000 hours, participants were asked to fast overnight. A total of 15 ml of clear CSF was collected in three polypropylene tubes labeled ‘A' (first 5 ml), ‘B' (second 5 ml) and ‘C' (third 5 ml). The tubes were immediately placed on ice for a maximum of 1 h until the samples were centrifuged at 4 °C (at 1500 r.p.m.) for 10 min. Then, aliquots of 0.25 ml were placed into 1.00-ml polypropylene cryogenic vials and put into Nunc eight-cell storage boxes (Nalge Nunc International, Rochester, NY, USA) at −80 °C. All amyloid-β, tau and amino-acid determinations were performed from tube ‘C'.

Among these participants, MDD patients (*N*=19) and comparison control subjects (*N*=17) were followed for 3 years. Fifteen individuals with MDD were receiving antidepressant treatment at the time of testing, but no differences in amyloid-β_42_ levels were observed within the MDD group as a function of antidepressant treatment.^26^ Clinical data, including physical examination, routine laboratory tests, psychiatric evaluations, HAM-D rating scale, cognitive functions and CSF samples, were collected at 3-year follow-up.

### Measurement of amino acids

This study was also approved by the Research Ethics Committee of the Graduate School of Medicine, Chiba University. Measurement of CSF levels of amino acids was carried out using high-performance liquid chromatography (HPLC) system (Shimadzu, Kyoto, Japan) as previously reported.^23, 25, 30^ Briefly, 15 μl of H_2_O (HPLC grade), 20 μl of 0.1 m borate buffer (pH 8.0) and 60 μl of 50 mm NBD-F in CH_3_CN (HPLC grade) were added into 5 μl of CSF sample. The reaction mixture was then heated at 60 °C for 2 min, and immediately supplemented with 100 μl of H_2_O/CH_3_CN (90/10) containing 0.1% trifluoroacetic acid to stop the reaction.

For determination of dl-serine, l-serine and d-serine, a 20-μl aliquot of the resultant solution was injected into the HPLC system.^25, 30^ A reversed-phase ODS column (TSKgel ODS-80Ts (Tosoh, Tokyo, Japan) as column 1) was used for the separation and quantification of total (d- and l-) serine, and the gradient elution of the mobile phase was maintained at a constant flow rate of 0.8 ml min^−1^. Mobile phase 1a consisted of H_2_O/CH_3_CN (90/10) containing 0.1% trifluoroacetic acid, and phases 1b and 1c, of H_2_O/CH_3_CN (10/90) containing 0.1% trifluoroacetic acid and CH_3_CN, respectively. The time program for gradient elution was as follows: 0–25 min 1a:1b:1c=92:8:0; 25–25.1 min linear gradient from 8% 1b to 100% 1b; 25.1–35 min 1a:1b:1c=0:100:0; 35–35.1 min linear gradient from 8% 1b to 100% 1c; 35.1–40 min 1a:1b:1c=0:0:100; and 40.1–60 min 1a:1b:1c=92:8:0. The chiral column (column 2) used for the separation and quantification of d- and l-serine with NBD-F comprised two Sumichiral OA-2500 columns (Sumika Chemical Analysis Service, Osaka, Japan), which were connected in tandem. The mobile phase was 15 mm citric acid in MeOH. The flow rate was isocratically pumped at 1.0 ml min^−1^. The column temperature of all columns was maintained at 35 °C. Fluorescence detection was performed at 530 nm, with an excitation wavelength at 470 nm.

For determination of glycine, glutamine and glutamate, a reversed-phase ODS column (TSKgel ODS-80Ts, Tosoh) was used.^23, 30^ The gradient elution of the mobile phase was kept at a constant flow rate of 0.8 ml min^−1^. The time program for gradient elution was programmed as follows: 0–50.5 min 1a:1b:1c=95:5:0; 50.5–55.5 min 1a:1b:1c=0:100:0; and 55.5–57 min 1a:1b:1c=0:0:100. The column temperature of all columns was maintained at 35°C. Fluorescence detection was performed at 530 nm, with an excitation wavelength at 470 nm.

### Statistical analysis

First, Student *t*- and *χ*^2^-tests were used to compare the MDD and comparison groups with respect to dimensions not associated with depression (that is, MMSE score, years of education, body mass index, age, incidence of diabetes, gender, *APOE* status and reported family history of AD; Table 1). Second, Student *t*-test was used to compare the two diagnostic groups with respect to all remaining CSF variables. Third, 3-year follow-up data were analyzed using a 2X2 repeated-measures analysis of variance; the factors were time (baseline and follow-up) and MDD status (MDD and controls). All tests were two-tailed, and statistical significance was established at an *α* of 0.05, unless differently noted. All analyses were conducted using SPSS 22 (SPSS, Chicago, IL, USA).

## Results

As reported previously,^26^ the two groups at baseline did not differ on any relevant clinical or demographic variable, with the exception of the mean HAM-D score, which was significantly higher in the MDD group (Table 1). Of note, the proportion of participants with a reported family history of AD was slightly higher in the comparison group than in the MDD group. The CSF levels of amyloid-β_42_ in the MDD group at baseline were significantly lower than those of the comparison group, whereas differences in CSF levels of amyloid-β_40_, and total and phosphorylated tau protein at baseline did not reach statistical significance across conditions (Table 1).^26^

There were no significant differences in levels of five amino acids (glutamine, glutamate, glycine, l-serine and d-serine) between the MDD (*N*=28) and comparison groups (*N*=19) at baseline (Table 2). However, the ratio of glutamine to glutamate in the MDD group was significantly higher than in the comparison group, whereas the ratio of glycine to l-serine and the ratio of d-serine to l-serine did not differ (Table 2). Furthermore, there were no correlations between CSF amino acids and typical AD biomarkers (amyloid-β_42_, amyloid-β_40_, and total and phosphorylated tau protein) at baseline in all subjects (data not shown).

**Figure 1:**
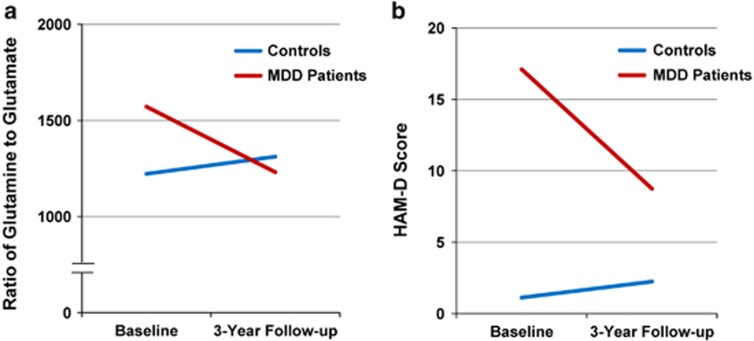
Three-year follow-up of elderly MDD patients and control comparison. (**a**) The ratio of glutamine to glutamate in the cerebrospinal fluid (CSF) from major depressive disorder (MDD) patient group (*N*=19) was significantly decreased at 3-year follow-up. In contrast, the ratio of glutamine to glutamate in the CSF from control comparison group (*N*=17) was slightly increased at 3-year follow-up. (**b**) The Hamilton Depression Rating Scale (HAM-D) score in the MDD patient group (*N*=19) was significantly decreased at 3-year follow-up. In contrast, HAM-D score in the control comparison group (*N*=17) was slightly increased at 3-year follow-up.

At the 3-year follow-up, an interaction between time and MDD status was observed for the HAM-D scores. *Post hoc* comparisons confirmed that the severity of depression decreased over time in participants with MDD (*P*=0.001), whereas no change was detected in controls (*P*=0.366; Table 3 and Figure 1). When the same repeated-measures analysis of variance was applied to CSF levels of amino acids, no further interactions or main effects were observed when setting *α* at 0.006 (Sidak correction; eight tests), although a trend was observed for an interaction with the glutamine/glutamate ratio (Table 3 and Figure 1). *Post hoc* comparisons suggested that there was a decrease in this ratio in MDD subjects (*P*=0.008), but no appreciable change in controls (*P*=0.571). Finally, we observed a marginally significant negative correlation (*r*=−0.328, *P*=0.051) between the change scores (baseline to follow-up) of the glutamine/glutamate ratio and of HAM-D scores in all subjects, suggesting that as depression severity decreased, so did the ratio.

## Discussion

In the present study, we found that older individuals with MDD showed an increased CSF glutamine/glutamate ratio compared with controls, although CSF levels of the individual amino acids were not different. To our knowledge, this is the first report in the literature of an increase in the glutamine/glutamate ratio in elderly MDD subjects, suggesting abnormalities in the glutamine–glutamate cycle in the brains of elderly depressed individuals. As the glutamine/glutamate ratio is an index for glutamine–glutamate cycle in the glia–neuron communication,^8, 23^ it is likely that abnormalities in the glutamine–glutamate cycle in the brain have a role in the pathophysiology of late-life depression.

We previously reported a reduction of amyloid-β_42_ in the same MDD patients at baseline.^26^ Importantly, the glutamine/glutamate ratio in elderly MDD patients was no longer significantly different from controls after a 3-year period; the loss of significance coincided with reduction in the severity of depressive symptoms, suggesting that abnormalities in this ratio in depression may be state- and not trait-dependent. However, we found no significant correlations between the ratio or levels of the individual amino acids and AD biomarkers, or severity of depressive symptoms either at the baseline or follow-up. These results are generally consistent with previous findings of CSF studies that also reported a lack of significant correlations between these amino acids and severity of depressive symptoms. Garakani *et al.*^22^ reported no significant differences between controls and MDD patients in baseline CSF levels of glutamate (*P*=0.761) or glutamine (*P*=0.226). Furthermore, there was a significant positive correlation (*r*=0.677, *P*=0.016) between intensity of suicidal ideation and CSF glutamate, and a significant negative correlation (*r*=−0.558, *P*=0.038) between baseline HAM-D score and the glutamate/glutamine ratio in adult patients (mean age of 40.4 years) with MDD. It is thus very difficult to reconcile the lack of significant correlations between the CSF ratio and severity of depressive symptoms with evidence that the increased CSF glutamine/glutamate ratio and other glutamatergic abnormalities associated with MDD might be state-dependent. Taken together, these findings highlight the limitations of static indices such as levels of glutamate or glutamine either in CSF or brain to reflect dynamic and brain region-specific alterations in the glutamatergic function. Using *in vivo* ^13^C-MRS and [1-^13^C]glucose in a single voxel spectroscopy study, Abdallah *et al.*^31^ found that a measure of the neuronal tricarboxylic acid cycle in the occipital cortex from medication-free MDD patients was 26% lower compared with controls, suggesting mitochondrial dysfunction in depression. However, they reported no significant alterations in the glutamine–glutamate cycle and γ-aminobutyric acid (GABA) synthesis, inconsistent with our CSF findings. Furthermore, this study did not find any correlations with severity of depression.^31^ One possibility is that the investigators focused only on the occipital cortex, and they did not examine CSF or other brain regions in which glutamatergic abnormalities have been found.

Glutamate is synthesized from glutamine by glutaminase, and metabolized to glutamine by the mainly astrocyte-located GS. In addition, glutamate is metabolized to GABA by glutamic acid decarboxylase (GAD).^8^ Released glutamate is taken up by surrounding astrocytes via the glutamate transporter, where it is converted to glutamine, transported back to presynaptic neurons and reconverted to glutamate. Thus, the glutamine–glutamate–GABA cycle as part of glia–neuron communication has an important role in excitatory and inhibitory neurotransmission.^8^ The expression of GS mRNA was reduced in the prefrontal cortex, premotor cortex and amygdala from depressed suicide subjects, but not in suicide completers without depression.^32, 33^ Furthermore, GS protein was decreased in the anterior cingulate cortex and orbitofrontal cortex from depressed patients.^32, 34, 35^ A recent study using postmortem brain samples showed that the density of GS-expressing astrocytes was significantly reduced in some cortical areas from MDD patients, suggesting a disturbance in the glutamine–glutamate cycle in MDD.^36^ Moreover, the immunoreactivity of EAAT2, one of the glutamate transporters in astrocytes, in the orbitofrontal cortex was significantly lower in MDD patients.^35^ In addition, increased immunoreactivity for GAD65/67 was demonstrated in neurons from several cortical regions, including orbitofrontal and dorsolateral prefrontal cortex, in depressed patients.^37^ By contrast, the amount of GAD67 in the dorsolateral prefrontal cortex of MDD patients was significantly reduced as compared with matched controls.^39^ These findings suggest a dysregulation of the glutamate–GABA cycle in depression. Taken together, it is likely that abnormalities in the glutamine–glutamate–GABA cycle in the brain have a role in the pathophysiology of depression.

In this study, we found that the glutamine/glutamate ratio in MDD patients was significantly decreased after 3-year follow-up in concert with improvement in depressive symptoms. Given the role of glutamatergic neurotransmission in the mechanisms of antidepressants,^5, 6^ it is noteworthy that symptom improvement caused by antidepressants may be mediated by effects on glutamine–glutamate cycle in the brains from depressed patients. In contrast, HAM-D score and the glutamine/glutamate ratio in healthy subjects were slightly increased after 3-year follow-up, although these increases were not statistically significant.

CSF levels of glutamine in individuals with AD were reported to be lower than in controls,^40^ whereas CSF levels of glutamate were higher.^42, 41^ Although the glutamine/glutamate ratio was not measured in either study, and depression was not a focus of either investigation, these results do not support a link between the abnormalities in the glutamine–glutamate cycle in depression with those in AD. Increased CSF levels of GS in individuals with AD compared with controls also have been reported.^43, 44^ In contrast, a recent study using a quantitative ELISA system showed that CSF levels of total GS in AD patients were not different from those observed in healthy controls,^38^ suggesting that total CSF levels of GS may not be a suitable biomarker for AD, or that further work needs to be done with this biomarker to clarify whether GS abnormalities exist in AD that are associated with the apparent abnormalities in mood disorders.

The NMDA-R antagonist ketamine is known to produce a rapid and sustained antidepressant effect in treatment-resistant patients with MDD.^11, 12^ A recent MRS study demonstrated that a single infusion of ketamine (0.5 mg kg^−1^) caused an increase of Glx and GABA in the medial prefrontal cortex of MDD patients in ~26 min,^45^ indicating that ketamine has a rapid effect on the glutamine–glutamate–GABA cycle in the prefrontal cortex in depressed patients. In contrast, earlier studies showed that a single infusion of ketamine (0.5 mg kg^−1^) did not produce changes in glutamate and glutamine concentrations in occipital cortex in MDD patients,^46^ or in the anterior cingulate/medial prefrontal cortex in healthy subjects,^47^ suggesting that changes in occipital amino-acid levels are not correlated with ketamine's antidepressant action. Thus, it would be of great interest to measure CSF amino acids in MDD patients at baseline and after a single ketamine infusion.

Finally, there are some limitations to this study that should be noted. The main limitation was small sample size, and similar, future studies in geriatric depression would likely benefit from larger sample sizes. Another limitation was the inability to measure CSF GABA in the subjects of this study. We could not measure CSF levels of GABA because these were below the lower limit of detection of our HPLC system. Given the key role of glutamine–glutamate–GABA cycle in glia–neuron communication,^5, 8^ it is of great interest to measure CSF GABA levels using a high-sensitive analytical system (for example, liquid chromatography coupled with tandem mass spectrometry).

In conclusion, we found that the CSF ratio of glutamine/glutamate levels in elderly patients with MDD was significantly higher than that of age-matched healthy controls, and that the increased ratio in patients was significantly decreased after 3-year follow-up by medication in conjunction with decreased depression symptoms over this time period. These findings suggest that abnormalities in the glutamine–glutamate cycle in the glia–neuron communication have a role in the pathophysiology of elderly depression. Further studies measuring CSF levels of amino acids (for example, glutamine, glutamate and GABA) using larger cohorts, particularly cohorts of antidepressant-naive patients, will be of great interest.

## Figures and Tables

**Table 1:** Demographic and clinical characteristics of cognitively intact individuals with MDD and age-matched comparison subjects at baseline

*Characteristic*	*Comparison group (*N=*19)*	*MDD group (*N=*28)*	*Statistical analysis*		
			t	*df*	P
Age (years)	68.1±7.3	66.5±5.4	0.835	45	0.41
Education (years)^a^	16.7±2.7	16.5±2.7	0.274	44	0.79
Body mass index	28.1±4.7	28.8±6.7	0.378	45	0.71
21-item HAM-D	1.2±1.9	14.9±8.8	8.02	45	**<0.001**
MMSE	29.5±0.5	29.8±0.6	1.56	45	0.13
Total recall rating	64.4±12.3	64.9±13.9	0.11	45	0.91
Delayed recall rating	8.5±2.8	9.5±2.5	1.258	45	0.22
					
*Trail-Making Test score*					
Part A	37.2±12.4	36.0±14.1	0.303	45	0.76
Part B	80.3 ±31.5	86.1±23.2	0.737	45	0.47
Category fluency test	41.7±8.1	40.6±8.2	0.45	45	0.66
					
	N *(%)*	N *(%)*	χ*^2^*	*df*	P
Diabetes	4 (21)	5 (18)	0.08	1	0.79
Female	12 (63)	10 (36)	2.41	1	0.12
					
*Apolipoprotein ɛ level*					
ɛ4	5 (26)	11 (39)	0.369	1	0.54
ɛ2	6 (32)	7 (25)	0.03	1	0.87
Family history of Alzheimer's disease	6 (32)	3 (11)	3.3	1	0.07
					
	*pg ml^−1^*	*pg ml^−1^*	t	*df*	P
Amyloid-β_42_	335.4±182.7	224.7±125.1	2.471	45	**0.02**
Amyloid-β_40_	6518.0±2687.0	5146.0±2369.0	1.845	45	0.07
Total tau protein^b^	328.7±151.7	273.0±114.3	1.422	44	0.16
Phosphorylated tau protein	51.6±20.9	48.9±25.9	0.371	45	0.71

Abbreviations: df, degrees of freedom; 21-item HAM-D, 21-item Hamilton Depression Rating Scale; MDD, major depressive disorder; MMSE, Mini-Mental State Examination. Bold *P*-values indicate statistically significant.

The data are mean±s.d. The data at baseline are from Pomara *et al.*^26^

a Data for one control subject were not available.

b Data for one MDD patient were not available.

**Table 2:** CSF levels of amino acids and ratio of amino acids in subjects at baseline

*Characteristic*	*Comparison group at baseline (*N=*19)*	*MDD group at baseline (*N=*28)*		*Analysis*	
			t	*df*	P
Glutamine	568.17±81.19	591.04±87.78	0.903	45	0.371
Glutamate	0.647±0.82	0.382±0.11	1.407	18.5^a^	0.176
Glycine	12.99±5.95	11.38±2.67	1.103	23.0^a^	0.282
l-Serine	25.45±5.58	22.36±5.13	1.955	45	0.057
d-Serine	1.76±0.38	1.75±0.43	0.093	45	0.926
Glutamine/glutamate	1257.6±458.4	1645.6±434.7	2.938	45	**0.005**^b^
Glycine/l-serine	0.496±0.14	0.526±0.14	0.7	45	0.488
d-Serine/l-serine	0.072±0.021	0.080±0.019	1.326	45	0.192

Abbreviations: CSF, cerebrospinal fluid; df, degrees of freedom; MDD, major depressive disorder.

Bold *P*-values indicate statistically significant. The data are mean±s.d.

a Equality of variance assumption not met.

b Denotes significant finding at *α*=0.006 after Sidak's adjustment (eight tests).

**Table 3:** HAM-D score and CSF levels of amino acids and ratio in subjects at baseline and 3-year follow-up

*Characteristic*	*Baseline*	*Baseline*	*3-year follow-up*	*3-year follow-up*	*Analyses (*P-*values)*		
	*Comparison group (*N=*17)*	*MDD group (*N=*19)*	*Comparison group (*N=*17)*	*MDD group (*N=*19)*	*ME time*	*ME MDD*	*Interaction*
21-item HAM-D	1.118±1.900	17.105±10.493	2.235±6.006	8.737±8.096	**0.005**	**<0.001**	**<0.001**
Glutamine	567.0±83.2	587.1±85.1	604.8±80.7	594.5±90.4	0.175	0.835	0.359
Glutamate	0.680±0.86	0.399±0.116	0.655±0.788	0.652±0.658	0.482	0.355	0.395
Glycine	13.30±6.18	11.96±2.79	13.2±4.51	13.37±5.20	0.456	0.668	0.388
l-Serine	25.8±5.68	23.23±5.69	25.0±5.66	25.4±7.79	0.564	0.537	0.207
d-Serine	1.76±0.40	1.75±0.45	1.80±0.41	1.91±0.49	0.09	0.717	0.315
Glutamine/glutamate	1221.98±472.7	1571.21±440.2	1311.06±463.9	1230.63±455.9	0.192	0.271	**0.029**
Glycine/l-serine	0.501±.147	0.536±0.153	0.546±0.208	0.535±0.128	0.467	0.787	0.454
d-Serine/l-serine	0.071±.021	0.078±0.021	0.074±0.018	0.079±0.021	0.277	0.41	0.673

Abbreviations: CSF, cerebrospinal fluid; 21 HAM-D, 21-item Hamilton Depression Rating Scale; MDD, major depressive disorder; ME MDD, main effect of MDD status (depressed and controls); ME time, main effect of time (baseline and follow-up).

The data are mean±s.d. The analysis method used was 2 × 2 repeated-measures analysis of variance. The bold values in the Analyses are statistically significant.
